# A Phosphotyrosine Switch in Estrogen Receptor β Is Required for Mouse Ovarian Function

**DOI:** 10.3389/fcell.2021.649087

**Published:** 2021-04-09

**Authors:** Bin Yuan, Jing Yang, Louis Dubeau, Yanfen Hu, Rong Li

**Affiliations:** ^1^Department of Biochemistry and Molecular Medicine, School of Medicine and Health Sciences, The George Washington University, Washington, DC, United States; ^2^Department of Pathology, USC/Norris Comprehensive Cancer Center, Keck School of Medicine of University of Southern California, Los Angeles, CA, United States; ^3^Department of Anatomy and Cell Biology, School of Medicine and Health Sciences, The George Washington University, Washington, DC, United States

**Keywords:** estrogen receptor beta, knock-in, ovary, follicle, fertility

## Abstract

The two homologous estrogen receptors ERα and ERβ exert distinct effects on their cognate tissues. Previous work from our laboratory identified an ERβ-specific phosphotyrosine residue that regulates ERβ transcriptional activity and antitumor function in breast cancer cells. To determine the physiological role of the ERβ phosphotyrosine residue in normal tissue development and function, we investigated a mutant mouse model (Y55F) whereby this particular tyrosine residue in endogenous mouse ERβ is mutated to phenylalanine. While grossly indistinguishable from their wild-type littermates, mutant female mice displayed reduced fertility, decreased ovarian follicular cell proliferation, and lower progesterone levels. Moreover, mutant ERβ from female mice during superovulation is defective in activating promoters of its target genes in ovarian tissues. Thus, our findings provide compelling genetic and molecular evidence for a role of isotype-specific ERβ phosphorylation in mouse ovarian development and function.

## Introduction

The steroid hormones estrogen and progesterone play vital roles in female reproduction, with the ovary serving as the primary tissue of their synthesis and one of the target sites (Emmen et al., 2005). The physiological actions of estrogen are mediated by two estrogen receptors: ERα and ERβ. Despite considerable sequence homology between these two ER isotypes, there is emerging evidence for their distinct and even opposite effects on their cognate cell and tissue types. In the ovary, ERα is the predominant ER isotype in theca and interstitial cells (Schomberg et al., 1999), ERα null females exhibit large, hemorrhagic and cystic follicles and infertility (Couse and Korach, 1999; Couse et al., 2003; Emmen and Korach, 2003). On the other hand, ERβ is predominantly localized in ovarian granulosa cells, which are the major source for ovarian steroid production and a major site of expression of luteinizing hormone (LH) receptor (Sar and Welsch, 1999; Pelletier et al., 2000). ERβ-mediated estrogen synthesis is important for follicle-stimulating hormone (FSH)-induced granulosa cell differentiation. Inactivation of ERβ in female mice leads to follicular maturation defect and subfertility (Krege et al., 1998; Dupont et al., 2000; Couse et al., 2005; Antal et al., 2008; Maneix et al., 2015).

The selective biological effects of ERα and ERβ partly result from their intrinsic differences in protein structure and transcriptional activity through recruitment of different transcription coactivators (O’Lone et al., 2004). Although the two ER subtypes share a high sequence homology in the central DNA binding domain and in carboxyl-terminal ligand-binding domain in the activation function domain 2, the more divergent sequence (∼15% sequence homology) in activation function domain 1 domain at the *N*-termini of the ER isotypes has been linked to subtype-specific activity (Madak-Erdogan et al., 2013). Both ERα and ERβ can directly bind to estrogen response elements (EREs) on DNA. In addition, they can exert transcriptional regulation by tethering to other transcription factors such as AP-1 and Sp1 on DNA (Saville et al., 2000; Cheung et al., 2005; Stender et al., 2010; Zhao et al., 2010). It is noteworthy that while 5% of ERβ-binding genomic regions contain ERE only, ∼60% of them include both ERE and AP-1 sites (Vivar et al., 2010; Zhao et al., 2010), suggesting that both ERE-binding and AP1-tethering mechanisms are involved in ERβ-dependent transcriptional activation. This may explain why earlier efforts to delete the DNA-binding domain of ERβ did not result in complete abolishment of ERβ function. Using a new ERβ knockout (KO) mouse line with deletion of all 10 exons of ERβ gene, a recent study found that ERβ regulates growth and differentiation of ventral prostate and mammary gland (Warner et al., 2020).

Despite considerable efforts to understand the role of ERβ posttranslational modifications (PTMs), the complexity of these modifications and their functions at the molecular, cellular, and organismal levels, are poorly understood (Tharun et al., 2015). So far, several phosphorylation residues, including Y36, S60, S75, S87, and S105, have been identified in human ERβ (Tremblay et al., 1999; Cheng et al., 2000; Tremblay and Giguere, 2001; St-Laurent et al., 2005; Picard et al., 2008; Lam et al., 2012; Yuan et al., 2014). In particular, our published work identified a subtype-specific phosphotyrosine residue in human ERβ Y36 that regulates its tumor-intrinsic (Yuan et al., 2014, 2016) and -extrinsic (Yuan et al., 2021) antitumor activity, which underscores an ER subtype-specific regulatory mechanism via tyrosine phosphorylation.

In this study, we used a mutant mouse strain carrying the equivalent of the human Y36 mutation in ERβ (*Esr2^*Y*55*F/Y*55*F*^*) to investigate the role of this phosphorylation residue in ERβ function in mouse ovaries. Mutant ovaries were smaller and showed diffusely luteinized stroma, compared to their WT counterparts. In addition, the reduced fertility phenotype of female KI mice was accompanied with lower circulating progesterone levels. Lastly, we provide molecular evidence for a role of this ERβ phosphotyrosine switch in regulation of steroidogenic transcription.

## Materials and Methods

### Animals

Genomic mutation of Y55F was introduced into the *Esr2* allele of mouse embryonic stem cells as described. WT and homozygous (*Esr2^*Y*55*F/Y*55*F*^*) mutant knock-in (KI) female littermates in the pure C57BL/6 background were generated by intercrossing of heterozygous *Esr2^+/Y55*F*^* mice. Mice were housed and maintained according to the Institutional Animal Care and Use Committee (IACUC) guidelines. Unless otherwise stated, mice were super-ovulated by intraperitoneal injection of 5 IU pregnant mare serum gonadotropin (PMSG, HOR-272, ProSpec), followed in 48 h with 5 IU of human chorionic gonadotropin (hCG, CG5, Millipore Sigma) for *in vivo* studies (Hong et al., 2010). An IACUC approved animal protocol was followed for all animal experiments.

### Western Blot Analysis

Ten isolated mouse ovaries at 8-week old were homogenized in Tissue Cell Lysis Buffer (GoldBio) with protease inhibitor cocktail (Roche). Protein concentration of cell lysates was determined using Pierce BCA Protein Assay Kits (#23225, Pierce). Primary antibodies against ERα (MC-20, Santa Cruz), ERβ (CWK-F12, Developmental Studies Hybridoma Bank and PPZ0506, Thermo Fisher), c-Abl (24-11, Santa Cruz), EYA2 (11314-1-AP, Proteintech), and β-actin (A5316, Sigma-Aldrich) and appropriate HRP-conjugated secondary antibodies were used for protein detection. Immunoblotting signals were visualized with ECL Western Blotting Substrate (Thermo Fisher).

### Subcellular Fractionation Assay

293T cells in 6 cm plates were transfected with 2 μg expression plasmids of either FLAG ERβ (WT) or ERβ (Y55F). Cells were harvested 24 h post transfection. NE-PER^TM^ Nuclear and Cytoplasmic Extraction Reagents (Thermo Fisher Scientific) were used to obtain nuclear and cytoplasmic fractions following manufacturer’s instructions. Subcellular fractionations were analyzed by Western blotting with α-Flag (F1804, Millipore Sigma) antibody to detect Flag-ERβ, and with α- H3 (Histone 3; # 9715, Cell Signaling), α-GAPDH (# 2118, Cell Signaling) antibodies as markers of the nuclear and cytoplasmic fractions, respectively.

### Immunoprecipitation

For evaluation of commercially available ERβ antibodies, immunoprecipitation (IP) was set up with 293T/Flag-mERβ cells using 4 μg each of anti-ERβ antibody, mouse and rabbit IgG (Vector Laboratories), anti-FLAG M2 Magnetic Beads (Millipore Sigma). The following ERβ antibodies were used in this assay: MC10, 51-7700 and PAI-311 (Thermo Fisher), CWK-F12 (Developmental Studies Hybridoma Bank), clone 9.88 and 68-4 (Millipore Sigma), H-150 (Santa Cruz), and EPR3777 (Novus).

### Immunohistochemistry

The anti-pY36 antibody, which was raised against the ERβ pY36-containing peptide SIYIPSS(pY)VDSHHE in rabbit (Yuan et al., 2014), and anti-Ki67 antibody (#12202, CST) were used. Immunohistochemistry (IHC) was performed as previously described (Yuan et al., 2014). Briefly, 5 μm paraffin-embedded sections were dewaxed in xylene, rehydrated, and processed for antigen retrieval with 10 mM citrate buffer (pH 6.0; Thermo Fisher). Tissue sections were subsequently incubated in 3% hydrogen peroxide for 10 min to quench endogenous peroxidase, and non-specific binding was blocked by incubation with normal goat serum (Vector Laboratories) for 30 min. Sections were then immunostained with anti-ERβ pY36 (1:50) and Ki67 (1:500) antibodies in PBS overnight at 4∘C. After washing, sections were incubated with a biotinylated goat anti-rabbit secondary antibody (1:200 dilution, Vector Laboratories) for 1 h at room temperature. Vectastain ABC kit (Vector Laboratories) was subsequently used for visualization according to the manufacturer’s instructions. The number of Ki67-positive granulosa cells from each ovary was recorded, and more than 1,500 cells from 7 individual animals were surveyed.

### Serum Hormone Analysis

For collection of mouse serum, mice were injected *i.p.* with 5 IU PMSG, followed by 5 IU hCG injection 48 h later. Mouse blood samples were collected 16 h after hCG injection. Serum hormone measurement was performed by the University of Texas Health San Antonio Institutional Mass Spectrometry Laboratory. Approximately 0.5 ml of blood was collected from each mouse, kept at 4∘C for 30 min, and then centrifuged at 6,000 *g* for 20 min. Serum samples were stored at −80∘C until processed for estradiol and progesterone measurements. For LC–MS/MS analyses, serum (100 μl made up to 200 μl with PBS), along with calibration standards (200 μl), and quality control samples (200 μl) were transferred into clean glass tubes and extracted with 1 mL of hexane:ethyl acetate (3:2 ratio) containing deuterated steroids as internal standards. Extracted samples were then left to allow phase separation at 4∘C for 1 h before placing them in a -80∘C freezer for 30 min to freeze the lower aqueous layer. The upper organic layer containing extracted target steroids was decanted into a clean glass tube and evaporated overnight at 37∘C. The dried samples were reconstituted in 1.2 mL of 20% methanol in PBS. After thorough mixing samples were transferred into 1.5 mL auto sampler vials and 1 mL was injected onto a C8 column for analysis. Steroid levels were calculated as amount per volume assayed for serum.

### Fertility Test

Fertility tests of female WT (*n* = 10) and KI (*n* = 10) mice were performed using continuous mating with male WT mice (Krege et al., 1998). Mating started when the mice were 8-week old and lasted for 6 months. The number of litters were counted and pups were recorded on postnatal day 1 (P1).

### Quantitative RT-PCR

Female mice were injected *i.p*. with 5 IU PMSG, followed by 5 IU hCG injection 48 h later. Ovarian samples were collected 16 h after hCG injection. Total RNA was reverse-transcribed using the ImProm-II^TM^ Reverse Transcription System (Promega). qPCR reactions were conducted in the ABI PRISM 7900HT Fast Real-Time PCR System using Luminaris Color HiGreen qPCR Master Mix (Thermo Fisher) and 0.25 μM of each primer, according to the manufacturer’s protocol. Primers for each gene are as follows:

*Cyp11a1* (F: 5′-AGGTCCTTCAATGAGATCCCTT-3′ and R: 5′-TCCCTGTAAATGGGGCCATAC-3′)
*Cyp19a1* (F: 5′-ATGTTCTTGGAAATGCTGAACCC-3′ and R: 5′-AGGACCTGGTATTGAAGACGAG-3′)
*PgR* (F: 5′-GGGGTGGAGGTCGTACAAG-3′ and R: 5′-GCGAGTAGAATGACAGCTCCTT-3′)
*Inhba* (F: 5′-TCACCATCCGTCTATTTCAGCA-3′ and R: 5′-CTTCCGAGCATCAACTACTTTCT-3′)
*Ptgs2* (F: 5′-TGAGCAACTATTCCAAACCAGC-3′ and R: 5′-GCACGTAGTCTTCGATCACTATC-3′)
*Lhcgr* (F: 5′-CGCCCGACTATCTCTCACCTA-3′ and R: 5′-GACAGATTGAGGAGGTTGTCAAA-3′).

### Chromatin Immunoprecipitation Assay

Chromatin immunoprecipitation (ChIP) was performed according to the procedures described previously (Yuan et al., 2014). Briefly, ovaries were submerged in PBS plus 1% formaldehyde, minced and incubated at room temperature for 15 min. Addition of 0.125 M glycine quenched the cross-linking reaction. Tissue pieces were then spun down and washed three times with ice-cold PBS. Chromatin was then isolated by adding lysis buffer, followed by disruption with a Dounce homogenizer. Lysates were sonicated to an average size of ∼200–500 bp using QSonica’s Q800R sonicator system (20% amplitude, 10 s on and 20 s off for 10 min). For each ChIP reaction, a total of 10 μg of antibody (PPZ0506, Thermo Fisher) was added to the precleared chromatin and incubated overnight at 4∘C. Subsequently, 50 μl of Dynabeads protein G (Invitrogen) was added to each ChIP reaction and incubated for 4 h at 4∘C. Dynabeads were washed with RIPA buffer (50 mM HEPES pH 7.6, 1 mM EDTA, 0.7% Na-deoxycholate, 1% NP-40, 0.5 M LiCl) three times and once with TE. Chromatin fragments were eluted, followed by reverse crosslinking and purified by phenol-chloroform extraction. ChIP DNA was resuspended in 10 mM Tris–HCl pH 8.5. Purified DNA was subjected to qPCR for assessing enrichment of specific genomic regions. Primers for each gene are as follows:

*PgR* (F: 5′-TCTACCCGCCATACCTTAACTA-3′ and R: 5′-CCAAAGACCAGCTCCACAA-3′)
*Inhba* (F: 5′-AGCCCAGAATATTCCACAGAAG-3′ and R: 5′-CTTTGGGAAAGCCTCCTCTC-3′)
*Ptgs2* (F: 5′-GGAAAGACAGAGTCACCACTAC-3′ and R: 5′-GAAGCTCTTAGCTCGCAGTT-3′)
*Lhcgr* (F: 5′-AGAGAAGCTTCCTCAGGTTTG-3′ and R: 5′-GGCTGTCTTTGTTATCCCAGTA-3′).

### Statistical Analysis

Statistical analysis was performed with PrismPad 7 software (GraphPad Prism Software Inc). Data are presented as mean ± SEM. Unless otherwise stated, statistical analyses was assessed using the two-tailed Student *t*-test assuming unequal variance. Threshold for statistical significance for each test was set at 95% confidence (*p* < 0.05).

## Results

### Establishment of an ERβ Y55 Mutant Mouse Strain

**FIGURE 1:**
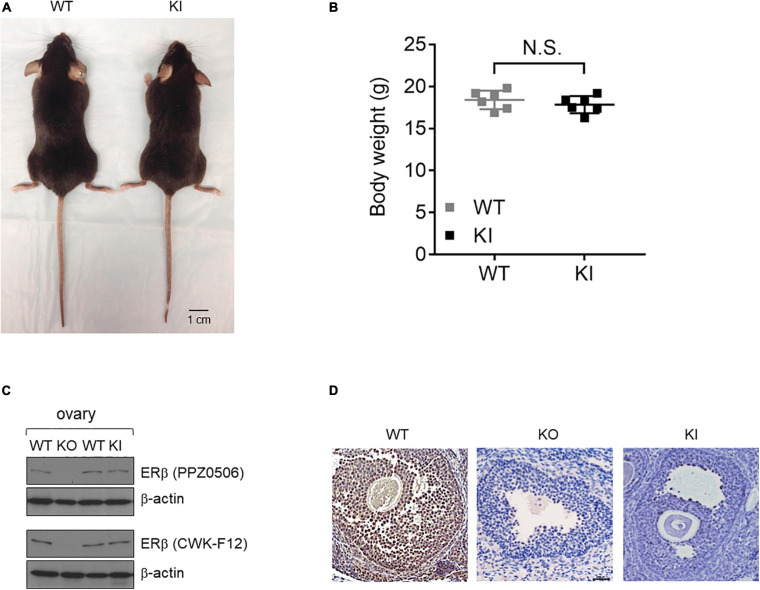
Generation and validation of the *Esr2* Y55F KI animal model. **(A)** The gross appearances of KI (right) and WT (left) female mice at 8 weeks of age. Scale bar, 1 cm. **(B)** WT and KI female mice body weight at 8-week old. Results are presented as mean ± SEM. *n* = 6; N.S.: Not Significant, Student’s *t*-test. **(C)** Western blot analysis of ovarian tissue extracts from ERβ KO, Y55F KI, and WT littermates. Two different commercial anti-ERβ antibodies were used. **(D)** Representative images of immunohistochemical staining for ERβ pY55 in the ovaries from WT, ERβ KO, and Y55F KI mice (*n* = 6 per genotype). Scale bar, 100 μm.

We used an ERβ Y55F whole body KI mouse line to investigate whether phosphorylation of site Y55 in mouse ERβ (Y36 in human, Supplementary Figure 1) plays a physiological role in ovarian development. Consistent with previously reported ERβ KO mice (Krege et al., 1998; Maneix et al., 2015), KI female mice had no obvious developmental defects and were indistinguishable in body size and weight from their WT littermates at 8-week old (Figures 1A,B, *n* = 6). For initial characterization of protein expression, we included a previously reported ERβ KO mouse model as control (Krege et al., 1998). ERβ protein expression in mutant ovaries was comparable to that in wild type ovaries based on results with two commercially available ERβ antibodies (Figure 1C). Levels of ERα, primarily expressed in theca cells, were not affected in KI ovaries (Supplementary Figure 2A). In addition, expression of c-Abl or EYA2, two upstream regulators of human ERβ Y36 phosphorylation (Yuan et al., 2014), was not altered either in KI or WT ovaries (Supplementary Figure 2A). Using an ERβ phosphotyrosine-specific antibody generated from our previous work (Yuan et al., 2014), we did not detect any appreciable IHC signal for phosphorylated ERβ in either KO or KI mutant mouse ovaries in contrast to the strong signals in wild type ovaries (Figure 1D). These results thus confirm genetic ablation of this particular tyrosine phosphorylation signaling in ERβ in KI ovarian tissue.

### The ERβ Phosphotyrosine Switch Is Important for Murine Ovarian Function

**FIGURE 2:**
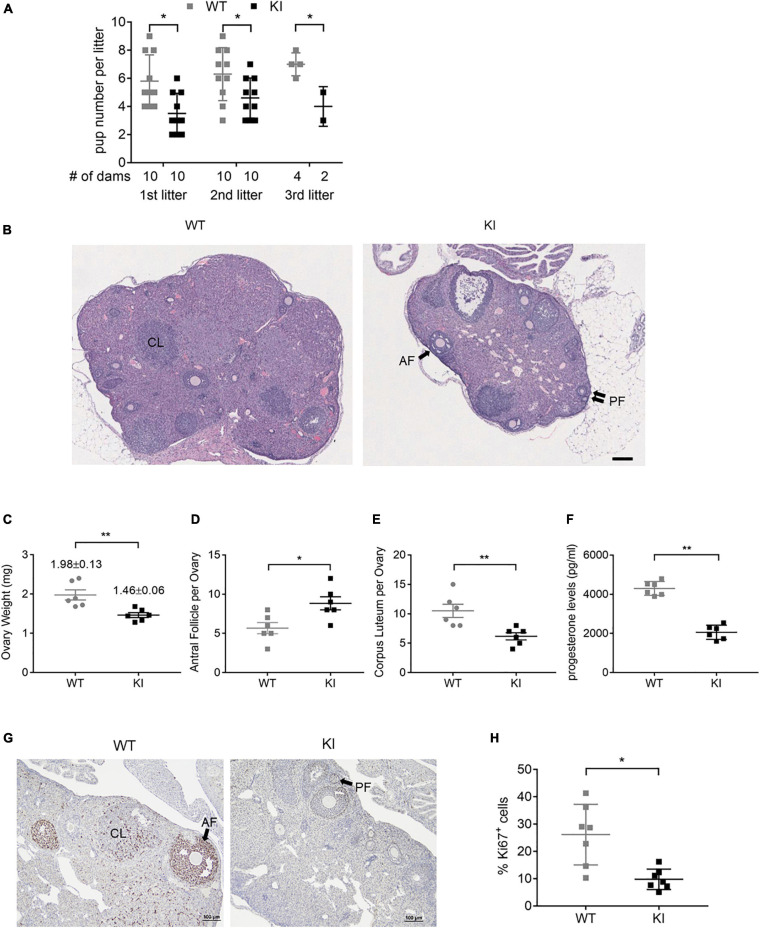
ERβ Y55 is important for murine ovarian functions. **(A)** Outcome of 6-month continuous mating for testing of female fertility. Results are presented as mean ± SD. **p < 0.05, Student’s *t*-test. **(B)** Representative H&E images of super-ovulated ERβ WT and KI ovaries. Scale bar, 100 μm. AF, antral follicle; PF, primary follicles; and CL, corpus luteum. **(C)** Mass of ovaries in WT and KI mice (*n* = 6) at the age of 8-week old. Values are presented as mean ± SEM. **p < 0.01, Student’s *t*-test. **(D, E)** Total number of antral follicle **(D)** and corpus luteum **(E)** per ovary from 6 ovaries of each genotype. Values are presented as mean ± SEM. *p < 0.05, **p < 0.01, and Student’s *t*-test. **(F)** Plasma level of progesterone from WT and KI female mice at 8–10 weeks of age (*n* = 6). Values are presented as mean ± SEM. **p < 0.01, Student’s *t*-test. **(G)** Representative images of immunohistochemical staining for the cell proliferation marker Ki67 in ovaries of WT and KI mice. Scale bar, 100 μm. **(H)** Quantification of Ki67-positive granulosa cells in the ovaries of WT and KI mice (*n* = 7). Values are presented as mean ± SEM. *p < 0.05.

To assess the impact of Y55F mutation on female fertility, we carried out continuous mating of WT and KI female mice with young mature WT male mice over a 6-month period. KI dams had substantially reduced litter sizes compared to WT control in each of the three pregnancies observed during the time period analyzed (Figure 2A), while the frequency of pregnancy was similar between WT and mutant groups (data not shown). On average, KI dams had 4.2 ± 0.4 pups per litter, versus 6.4 ± 0.4 for WT (*p* < 0.01). The reduced fertility phenotype of KI mutant is consistent with previously reported fertility defect of *Esr2* KO mice (3.1 ± 1.8 pups per litter; Krege et al., 1998). To further interrogate functional deficiency of KI mice, we treated age-matched WT and KI female littermates with PMSG and hCG consecutively to achieve superovulation. KI ovaries were smaller than their wild type counterparts, showed a diffusely luteinized stroma, and had significantly more antral follicles and fewer corpora lutea that were less demarcated from surrounding stromal cells compared to wild type ovaries (Figures 2B–E, *n* = 6). There was no statistical difference in hemorrhagic follicles between WT and KI ovaries by total count (Supplementary Figure 2B).

The ovary is the primary source of estrogen and progesterone synthesis in fertile females. As a measurement of this aspect of ovarian function, hormone concentrations in plasma serum were compared in WT and KI female mice. KI mice had normal circulating estrogen levels (Supplementary Figure 2C) but significantly lower progesterone levels versus WT (Figure 2F), which is consistent with the reduced litter size of KI dams. In addition, ovaries of KI mice displayed lower percentages of Ki67-positive granulosa cells compared to WT controls (Figures 2G,H, *n* = 7), indicating reduced cell proliferation.

### Y55 Is Important for ERβ-Dependent Transcription in Ovarian Tissue

**FIGURE 3:**
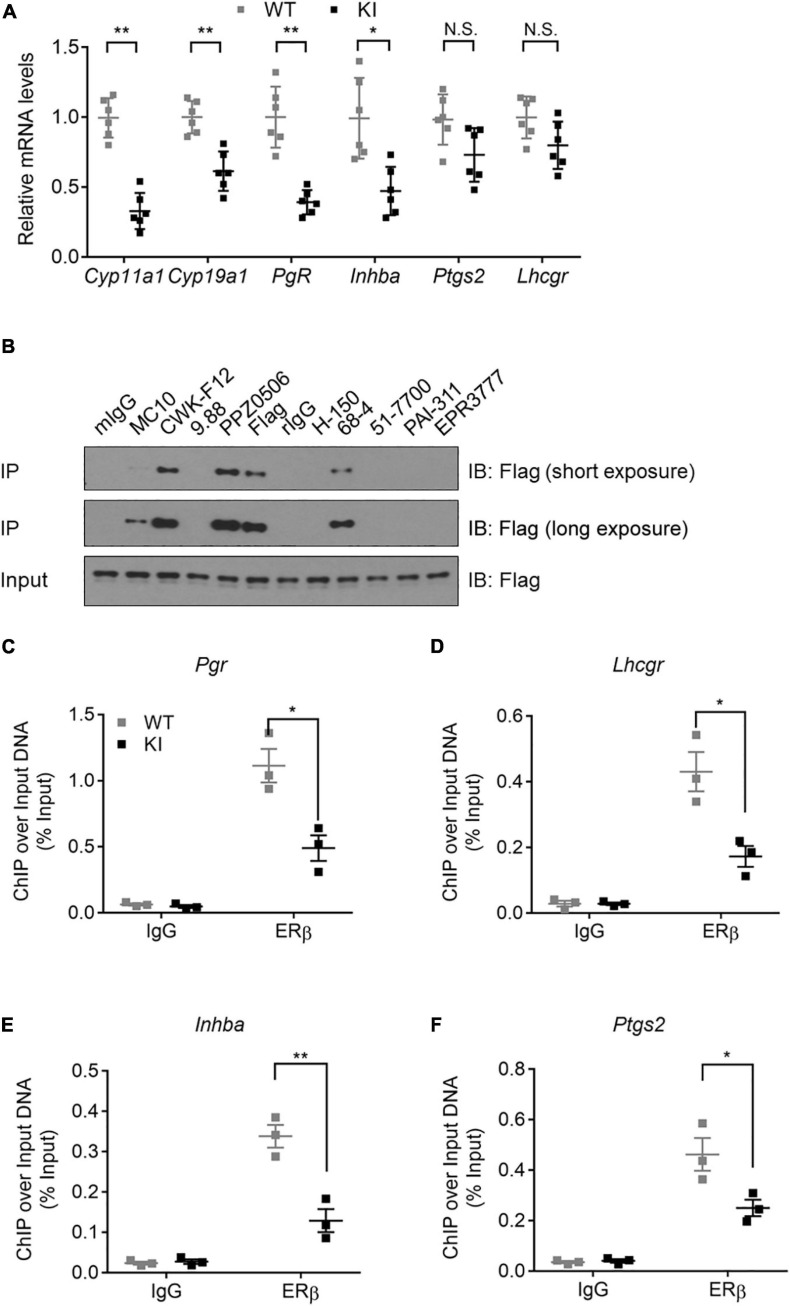
Y55 is important for ERβ transcriptional activity in murine ovaries. **(A)** Levels of mRNA for steroidogenesis genes in the ovaries from WT and KI mice (*n* = 6) after superovulation. N.S.: Not Significant, **p < 0.01, *p < 0.05, and Student’s *t*-test. **(B)** IP-WB of 293T/Flag-mERβ cells with a panel of commercial anti-ERβ antibodies. **(C–F)** ChIP-qPCR analyses of ERβ occupancy near TSS of the *Pgr* **(C)**, *Lhcgr* **(D)**, *Inhba* **(E)**, and *Ptgs2* **(F)** genes in mouse ovaries from WT and KI mice. **p < 0.01, *p < 0.05, and Student’s *t*-test.

To determine the molecular defect associated with the Y55F mutant, we first asked whether Y55F mutation affects the relative abundance of ERβ in cytoplasm versus nucleus. Levels of Flag-tagged ERβ WT and Y55F protein were comparable in both cytoplasmic and nuclear fractions (Supplementary Figure 2D). Next, we analyzed mRNA levels of several key steroidogenesis-related ERβ target genes in ovarian tissues of WT and KI mice. We found that KI mice exhibited reduced expression of *Cyp11a1*, *Cyp19a1*, progesterone receptor *(Pgr)*, and *Inhba* as compared to their WT counterparts (Figure 3A). Levels of prostaglandin-endoperoxide synthase 2 (*Ptgs2)* and *Lhcgr* mRNA were also reduced in KI ovaries but the changes were not statistically significant (Figure 3A). We then determined the impact of the Y55F mutation on ERβ chromatin binding to its target transcription promoters. Due to the concerns over the specificity and IP efficiency of commercially available anti-ERβ antibodies (Weitsman et al., 2006; Andersson et al., 2017; Nelson et al., 2017), we screened a panel of commercial anti-ERβ antibodies for their ability to IP FLAG-tagged mouse ERβ in human 293T cells (ERβ-negative). We found that anti-ERβ antibodies CWK-F12 and PPZ0506 gave rise to robust and specific ERβ IP signals (Figure 3B). Our result is consistent with a recent report regarding specific cross-reactivity of PPZ0506 against human and mouse ERβ proteins (Ishii et al., 2019). ChIP indicates that ERβ chromatin occupancy at the promoter regions of its target genes is substantially reduced in KI mutant ovaries (Figures 3C–F). Taken together, these data strongly support the notion that phosphotyrosine-dependent ERβ signaling regulates ERβ-dependent steroidogenic transcription program in mouse ovaries.

## Discussion

As nuclear hormone receptors, ERα and ERβ are involved in regulation of many complex physiological processes in humans and rodents (Paterni et al., 2014). For example, ERα promotes cell proliferation in an estrogen-dependent manner in the breast, whereas ERβ inhibits proliferation of multiple cancer cell types including breast, prostate and colon while facilitating granulosa cell differentiation during ovarian folliculogenesis. In contrast to the extensive literature on ERα function, how ERβ biological activity is regulated remains vastly under-explored. In the current study, we used a mouse genetic model to demonstrate a definitive role of an ERβ-specific phosphotyrosine switch in regulation of mouse ovarian functions including fertility, steroidogenesis, and ovarian transcription. However, we point out that our current study did not investigate the effect of ERβ Y55F mutation on the hypothalamus-pituitary axis. Therefore, the ovarian defects observed in whole-body KI mice could be contributed by lower gonadotropin secretion and subsequent reduction in follicle growth due to hypothalamus-pituitary dysfunction.

Three animal models for ERα phosphorylation site mutants were reported recently. Female mice carrying a mutant S122 ERα had a tissue-preferential impact on adipose tissue mass (Ohlsson et al., 2020). In a separate report, blocking of ERα Ser216 phosphorylation aggravated microglia activation and brain inflammation (Shindo et al., 2020). In a third study, dramatic developmental defects in the reproductive organs, mammary glands, and bones were observed in a Cre-inducible mouse model carrying an ERα Y541 mutation (ERα Y537S in humans; Simond et al., 2020). The ovarian phenotype of ERβ Y55F KI mice described in the current study extends the accumulating genetic evidence for the distinct impact of various PTMs on the physiological functions of the two ER isotypes in multiple tissues and organs.

In a previous study, loss of ERβ was shown to cause spontaneous granulosa cell tumors as well as pituitary tumors in 20 to 24 month-old female animals (Fan et al., 2010). The elevated incidence of pituitary tumors in ERβ KO mice was likely due to excessive secretion of gonadotropin releasing hormone from hypothalamus. No increased tumor incidence was observed in our Y55F KI mice up to 17 months of age. However, using transplant tumor models, we found that KI mice experienced compromised antitumor immunity and accelerated tumor growth (Yuan et al., 2021), suggesting a tumor-suppressive activity of host ERβ signaling.

ERβ, but not ERα, is known to be highly expressed in ovarian granulosa cells (Schomberg et al., 1999; Inzunza et al., 2007; Ishii et al., 2019). In response to gonadotropins, ERβ promotes transcriptional activation of multiple differentiation/steroidogenic genes including *Cyp11a1*, *Cyp19a1*, and *Lhcgr*. *Cyp11a1* encodes the cytochrome P450 cholesterol side-chain cleavage (P450scc) enzyme, which catalyzes conversion of cholesterol to pregnenolone (Hu et al., 2004; Chien et al., 2013). This is the first step in steroidogenesis that specializes in steroid hormone production. *Cyp19a1* encodes aromatase, an important enzyme in the aromatization of androgen to estrogen (Hanukoglu, 1992) and the rate-limiting step in estrogen biosynthesis. In addition, ERβ is required for expression of known LH-responsive genes such as *Ptgs2* and *Pgr* (Couse et al., 2005; Emmen et al., 2005). Our mRNA analysis clearly shows that ERβ-dependent transcription of these steroidogenic genes is regulated by the Y55 phosphotyrosine switch. Our published work identifies c-Abl and EYA2 as the corresponding kinase and phosphatase that directly control the phosphorylation status of this molecular switch in human breast cancer cells (Yuan et al., 2014). Future work is needed to determine whether the same kinase/phosphatase pair is involved in regulation of ERβ functions via Y55 phosphorylation in mouse ovaries.

Due to technical limitation, whole ovaries instead of purified primary granulosa cells were used in our ERβ ChIP analysis. We recognize that reduced granulosa cell proliferation in KI ovaries could complicate the interpretation of our ERβ ChIP result. It will be important to confirm dynamics of ERβ chromatin binding in purified granulosa cell populations using more sensitive ChIP conditions.

Not all granulosa cell-related genes examined were down-regulated in KI ovaries. For example, *Ptgs2* and *Lhcgr* mRNA levels were not significantly different in WT and KI ovaries. This could be due to the following reasons. LH transiently up-regulates *Ptgs2* in granulosa cells of large antral follicles within 4 h (Carletti and Christenson, 2009), but its effect on *Ptgs2* mRNA expression after 11 h differs in different studies (Joyce et al., 2001; Lee-Thacker et al., 2020). In addition, PTGS2 protein is also expressed in the interstitial cells of rat ovaries (Quintana et al., 2008). Regarding *Lhcgr* mRNA expression, it is known that FSH induces *Lhcgr* only in mural granulosa cells (Erickson et al., 1979; Law et al., 2013), which do not proliferate in response to FSH. Thus, the impact of granulosa cell growth defects on *Lhcgr* mRNA levels is likely to be less than on those genes that are uniformly expressed in granulosa cells. Additionally, *Lhcgr* is down-regulated in preovulatory granulosa cells and corpus luteum. Thus, the post-superovulation treatment may not be the best way to evaluate *Lhcgr* levels in KI ovaries.

Our previous cancer-related study shows that the ERβ-specific agonist S-equol elevates the same phosphotyrosine switch in cancer cells and antitumor CD8^+^ T cells and inhibits tumor growth in both xenograft and syngeneic tumor models (Yuan et al., 2016, 2021). S-equol is a natural compound with proven clinical safety and high tolerance in humans, based on multiple phase I and II clinical trials (Setchell et al., 2005; Jackson et al., 2011; Takeda et al., 2018). Our finding of the importance of this ERβ molecular switch in supporting ovarian functions raises the distinct possibility that S-equol could be explored for potential treatment of infertility and other related reproductive disorders.

## Data Availability Statement

The original contributions presented in the study are included in the article/Supplementary Material, further inquiries can be directed to the corresponding author/s.

## Ethics Statement

The animal study was reviewed and approved by Institutional Animal Care and Use Committee (IACUC), The George Washington University.

## Author Contributions

RL conceived and supervised the project. RL and YH designed the experiments. BY, JY, and LD performed the experiments and analyzed the data. RL and BY wrote the manuscript. All authors contributed to the article and approved the submitted version.

## Conflict of Interest

The authors declare that the research was conducted in the absence of any commercial or financial relationships that could be construed as a potential conflict of interest.
