# Supplementary figures and images for: A Phosphotyrosine Switch in Estrogen Receptor β Is Required for Mouse Ovarian Function
Source: Front Cell Dev Biol. 2021 Apr 9;9:649087. doi: 10.3389/fcell.2021.649087 (PMC8063698; doi:10.3389/fcell.2021.649087)

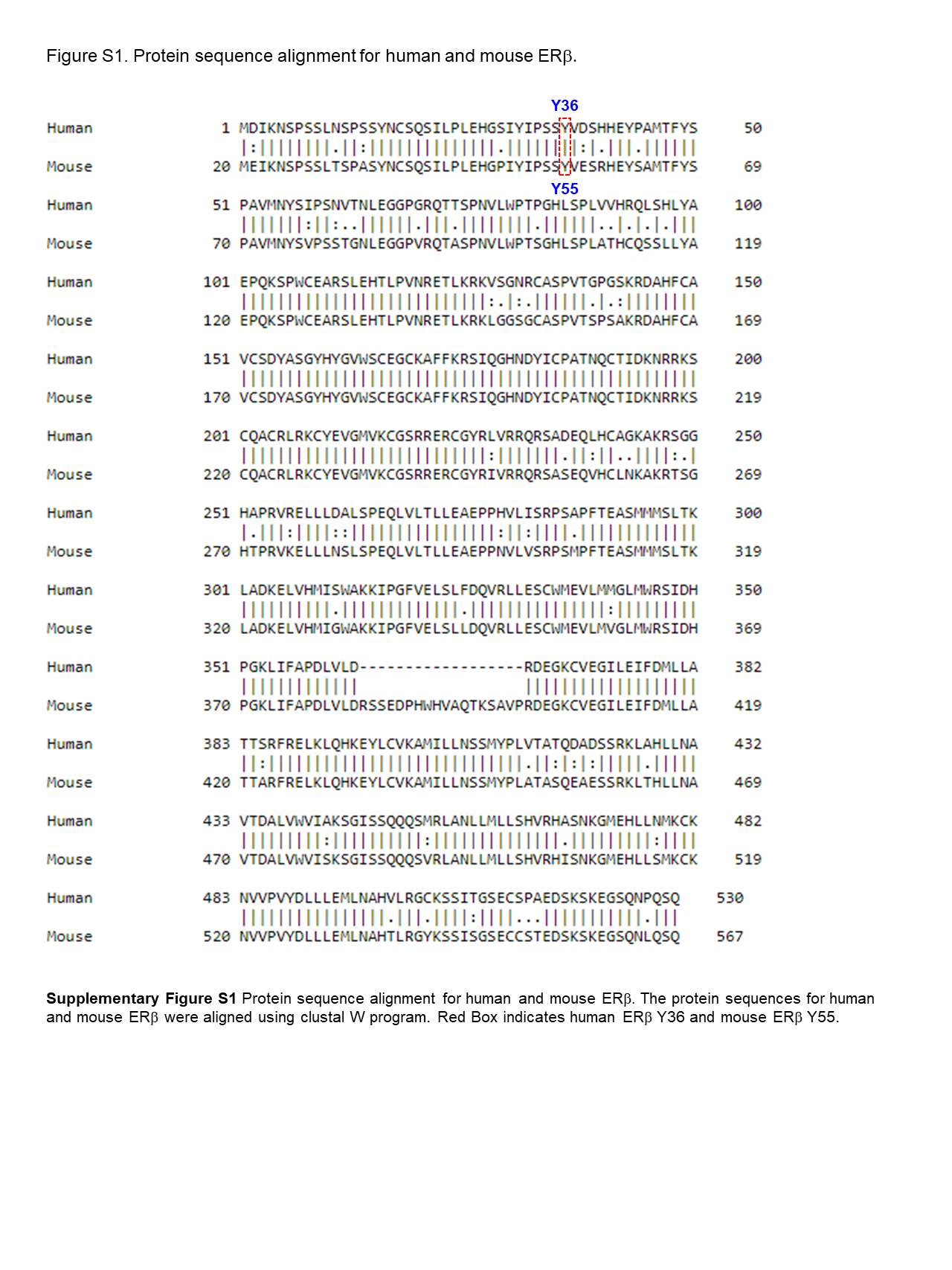

Supplement: Supplementary file 1 [file Image_1.TIF]

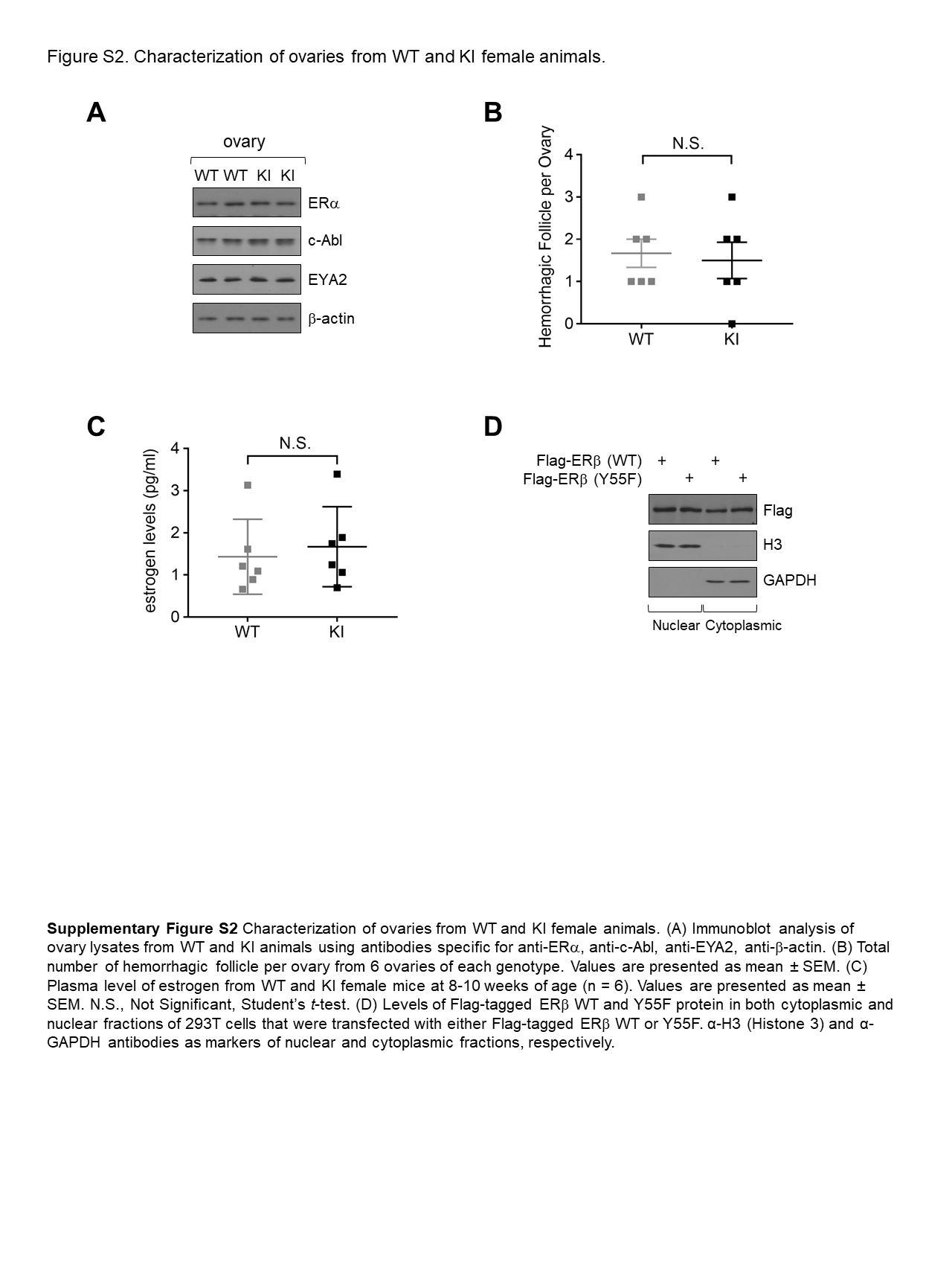

Supplement: Supplementary file 2 [file Image_2.TIF]
